# Simultaneous measurements of HERFD-XANES, RXES and RIXS of caesium using a transition-edge sensor

**DOI:** 10.1107/S1600577526001682

**Published:** 2026-04-03

**Authors:** Akiko Yamaguchi, Shinya Yamada, Tadashi Hashimoto, Takuma Okumura, Yasuo Takeichi, Masahiko Okumura, Yoshio Takahashi

**Affiliations:** a Center for Computational Science and e-Systems Japan Atomic Energy Agency Kashiwa Chiba277-0871 Japan; b Department of Earth and Planetary Science, Graduate School of Science The University of Tokyo Bunkyo Tokyo113-0033 Japan; c Department of Physics Rikkyo University Toshima Tokyo171-8501 Japan; d RIKEN Wako Saitama351-0198 Japan; e Advanced Science Research Center Japan Atomic Energy Agency Tokai Ibaraki319-1195 Japan; f Tokyo Metropolitan University Hachioji Tokyo192-0397 Japan; g Institute of Materials Structure Science High Energy Accelerator Research Organization 1-1 Oho Tsukuba Ibaraki305-0801 Japan; ESRF – The European Synchrotron, France

**Keywords:** caesium, transition-edge sensor (TES), X-ray absorption near-edge structure (XANES), resonant inelastic X-ray scattering (RIXS), high-energy resolution fluorescence-detected XANES (HERFD-XANES)

## Abstract

A transition-edge sensor (TES) enables high-resolution measurements of X-ray fluorescence across a wide energy range, allowing for the simultaneous acquisition of high-energy resolution fluorescence-detected X-ray absorption near-edge structure (HERFD-XANES) spectra, resonant X-ray emission spectroscopy (RXES) spectra and resonant inelastic X-ray scattering (RIXS) planes in a single scan. Herein, we employed a TES to investigate various caesium compounds. The results suggest that HERFD-XANES, RXES and RIXS measurements *via* a TES can effectively reveal detailed information on the chemical states of the target element. This study also compares the performance of the TES with that of a crystal analyzer spectrometer. Although the two techniques differ in achievable energy resolution, the TES provides several distinct advantages, including easier installation, no analyzer exchange, faster measurements and simultaneous detection of multiple emission lines.

## Abstract 2

A transition-edge sensor (TES) enables simultaneous measurements of HERFD-XANES, RXES and RIXS with a high-energy resolution. This study employed a TES to investigate caesium compounds at the Cs *L*_II_-edge (5359 eV).

## 1. Introduction

X-ray absorption near-edge structure (XANES), a component of X-ray absorption fine structure, arises from the excitation of inner-shell electrons and is a powerful tool for investigating the chemical species, electronic states and local structures of target elements. Its high selectivity and sensitivity are particularly advantageous for analyzing environmental samples, which often contain many impurities and low concentrations of the target element. Although the most com­mon method for obtaining a XANES spectrum is transmission mode – where absorption is measured using two detectors placed before and after the sample – the XANES spectrum can also be acquired in fluorescence mode, which detects the intensity of emitted X-ray fluorescence (XRF). The fluorescence mode is particularly effective for samples containing low concentrations of the target elements. In many cases, the intensity of XRF is measured using a solid-state detector (SSD) or a silicon drift detector (SDD), both of which provide sufficient energy resolution to selectively detect the emission lines corresponding to the element of interest.

In recent decades, technologies for detecting X-rays with high-energy resolution have advanced significantly, enabling the acquisition of XANES spectra with sharp and distinct features, known as high-energy resolution fluorescence-detected XANES (HERFD-XANES) (Hämäläinen *et al.*, 1991 ▸; Glatzel *et al.*, 2013 ▸; Asakura & Tanaka, 2021 ▸). These measurements provide detailed spectral information and make it possible to identify subtle differences between chemical compounds. A well-established method for high-resolution XRF detection is the wavelength-dispersive crystal analyzer spectrometer (CAS), which achieves an energy resolution of <4 eV in full width at half maximum (FWHM) – far superior to the 150–500 eV resolution typically offered by SSDs and SDDs (Proux *et al.*, 2017 ▸; Asakura & Tanaka, 2021 ▸). HERFD-XANES measurements using a CAS have successfully identified the chemical species of highly diluted trace elements with remarkable precision (Proux *et al.*, 2017 ▸; Kona­gaya *et al.*, 2021 ▸; Natori *et al.*, 2022 ▸; Bazarkina *et al.*, 2025 ▸). However, a notable limitation of the CAS is that its detectable X-ray energy is restricted to a single configuration. When the energy of the focused X-ray shifts by a certain amount (*e.g.* ∼250 eV), the analyzer crystals must be replaced and finely realigned to accommodate the new target energy.

Herein, we propose that the TES is a promising alternative, functioning as a high-efficiency energy-dispersive X-ray detector. A TES measures X-ray energy with high resolution by detecting changes in the resistance and temperature of superconducting materials upon X-ray absorption. Its energy resolution is ∼5 eV at ∼6 keV X-rays (Yamada *et al.*, 2021 ▸), which, although not as high as that of CAS, is much better than that of SSDs and SDDs. The high resolution enables the distinction of XRF with closely spaced energies, allowing XANES spectra to be obtained with samples containing low concentrations of the target element and severe spectral interference from coexisting elements – conditions under which SSDs and SDDs fail. For example, our group has successfully acquired uranium *L*_III_-edge XANES spectra in rubidium-rich samples (Yomogida *et al.*, 2024 ▸), cerium *L*_II_-edge XANES spectra in titanium (Ti)-rich samples (Li *et al.*, 2023 ▸) and caesium (Cs) *L*_III_-edge XANES spectra in Ti-rich samples (Takahashi *et al.*, 2025 ▸).

Another advantage of the TES is its wide detectable energy range within a setup – exceeding 2 keV – which allows for the simultaneous acquisition of HERFD-XANES and resonant X-ray emission spectroscopy (RXES). X-ray emission spectroscopy (XES), which arises from inner-shell electron excitations followed by fluorescence emission, provides insights into the chemical species and electronic states of target elements. While XES can be per­for­med using non-monochromatic sources such as X-ray tubes, RXES refers specifically to XES per­for­med with a tuned incident X-ray energy and involving resonance (Joly & Grenier, 2015 ▸). Because the theoretical basis of XES differs from that of XANES, the two techniques are complementary. For example, combining XES and XANES measurements allows for the clear differentiation of Ti species in certain compounds (Reinhardt *et al.*, 2009 ▸).

Uhlig *et al.* (2015 ▸) provided a detailed discussion of the energy resolution of various energy- and wavelength-dispersive detectors and demonstrated the capability of the TES for both soft and hard X-ray XES under off-resonant excitation. Typically, high-resolution measurements of both XANES and XES require multiple facilities and experimental setups. However, the TES enables both measurements to be per­for­med simultaneously in a single experiment. In addition, the TES can collect resonant inelastic X-ray scattering (RIXS) planes within a single XANES scan. RIXS has attracted considerable interest as a powerful technique for probing the fundamental excitations of target elements (de Groot *et al.*, 2024 ▸). The RIXS planes covering multiple emission lines can also be acquired in a single scan, owing to the wide detectable energy range of the TES.

To evaluate the capabilities of the TES, we chose the Cs *L*_II_-edge as our target, primarily because (i) its energy lies in the tender X-ray region and (ii) the TES offers better energy resolution at lower photon energies. The TES is a microcalorimeter that measures the total deposited photon energy as a temperature rise in a superconducting film operated within its resistive transition. As a result, higher energy resolution is generally achieved at lower photon energies, while partial saturation effects become more significant at higher photon energies (Yamada *et al.*, 2021 ▸; Gottardi & Smith, 2023 ▸). Although TES-based measurements have been demonstrated in the soft X-ray region, to the best of our knowledge, no studies have reported measurements at emitted X-ray energies exceeding 2 keV. In addition, Cs is of particular interest owing to (i) its importance in environmental chemistry and (ii) its unique chemical behaviour. It easily forms the monovalent ion Cs^+^ and generally exhibits ionic characteristics similar to other alkali metals. However, according to the hard and soft acids and bases (HSAB) theory (Pearson, 1963 ▸), Cs^+^ is classified as a soft acid and covalent bonding involving Cs – particularly under high-pressure conditions – has been reported (Miao, 2013 ▸). These behaviours are likely associated with its exceptionally large ionic radius, which affects various chemical reactions. Cs is a key element in environmental chemistry because radioactive isotopes, such as ^137^Cs and ^134^Cs, have been released into the environment through nuclear accidents (Sansone *et al.*, 1996 ▸; McKinley *et al.*, 2001 ▸; Takahashi *et al.*, 2024 ▸), and they are also important in the context of the geological disposal of radioactive waste (Langmuir, 1997 ▸). In addition, Cs plays an important role in materials chemistry, particularly in applications such as gas sensors (Kumar *et al.*, 2024 ▸). Therefore, a reliable method for determining the chemical species and electronic states of Cs is valuable across multiple fields. In this context, this study aims to investigate the chemical characteristics of Cs in selected compounds using HERFD-XANES, RXES and RIXS spectra acquired with the TES. Although the Cs *L*_III_-edge is com­monly employed for Cs *L*-edge spectroscopy, the *L*_II_-edge was selected in this study to enable normalization of the XES and RIXS spectra by the intensity of the Cs *L*α emission line, which is simultaneously measured with the TES.

## 2. Methods

Caesium compounds, including caesium nitrate (CsNO_3_), caesium sulfate (Cs_2_SO_4_), caesium bromide (CsBr) and caesium iodide (CsI), were mixed with boron nitrate to form pellets with a Cs concentration of ∼200 ppm. In addition, a solution sample containing 0.2 m*M* Cs^+^ was prepared by dissolving CsNO_3_ in ultrapure water to represent hydrated Cs^+^.

**Figure 1:**
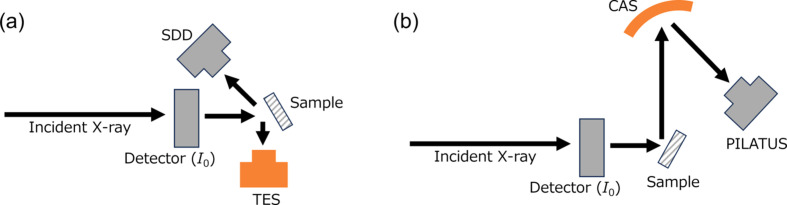
Schematic diagrams of the experimental setups for (*a*) simultaneous HERFD-XANES and XES measurements using the TES, and (*b*) HERFD-XANES measurements using the CAS.

Simultaneous measurements of HERFD-XANES and RXES using the TES were per­for­med at beamline BL37XU at SPring-8 (Hyogo, Japan). The incident X-rays were monochromated using an Si(111) double-crystal monochromator with an energy resolution of Δ*E*/*E* = 2 × 10^−4^, and the emitted XRF was detected using a TES detector comprising a 240-pixel array with an active area of 23.4 mm^2^. The TES was operated at temperatures ranging from approximately 70 to 80 mK. The sensor energy cutoff and saturation energy were both approximately 18 keV. The experimental setup was essentially identical to that described by Yamada *et al.* (2021 ▸), except that, in the present study, a collimator was placed immediately upstream of the TES array to suppress scattered X-rays incident on the detector. A single simultaneous measurement of HERFD-XANES and RXES required ∼1 h. During the measurements, the XRF was also detected by an SDD for comparison. To ensure reproducibility, CsNO_3_ was measured twice. For comparison, HERFD-XANES measurements using a CAS were conducted at beamline BL39XU in SPring-8. An Si(220) double-crystal monochromator with an energy resolution of Δ*E*/*E* = 1 × 10^−4^ was used for the incident X-rays and Ge(400) spherically bent crystals were employed for analyzing the emitted X-rays. Detailed conditions are provided elsewhere (Kawamura, 2020 ▸; Kawamura & Higashi, 2024 ▸). The experimental setups are illustrated in schematic diagrams (Fig. 1 ▸). The TES and SDD were located close to the sample [Fig. 1 ▸(*a*)], while the CAS and PILATUS 100K detector were positioned on the Rowland circle of 820 mm diameter and precisely aligned for each target fluorescence X-ray energy during XES scans [Fig. 1 ▸(*b*)].

XANES spectra were normalized and analyzed using standard procedures. The centroid energies of the white lines were determined by fitting the spectra with Gaussian and arc­tangent functions using *NumPy* (Version 1.26.4) and the ‘curve_fit’ function from *SciPy* (Version 1.13.1) in Python (Version 3.9.7). The RXES spectra were fitted with Voigt functions, defined as the convolution of Gaussian and Lorentzian profiles, using ‘voigt_profile’ and the ‘curve_fit’ functions from the *SciPy* library. Additional dependencies in­cluded *pandas* (Version 2.2.3) and *Matplotlib* (Version 3.9.2).

XANES simulations of CsBr were per­for­med using the *WIEN2k* program package, which implements density functional theory (DFT) based on the full-potential (linearized) augmented plane-wave [(L)APW] + local orbitals (lo) method (Blaha *et al.*, 2020 ▸). A 4 × 4 × 4 supercell was used and the muffin-tin radii were set to 2.5. The RKmax parameter was set to 6.0, and a 14 × 14 × 14 Monkhorst–Pack (Monkhorst & Pack, 1976 ▸) *k*-point mesh was used. The spectrometer broadening and the core-hole lifetime broadening were 0.1 and 0.75, respectively. The crystal structure of CsBr used in the simulations was geometrically optimized using the *Vienna Ab initio Simulation Package* (*VASP*) (Kresse & Furthmüller, 1996 ▸) with the Perdew–Burke–Ernzerhof exchange-correlation functional (Perdew *et al.*, 1996 ▸). The projector-augmented wave (Blöchl, 1994 ▸) potentials from the *VASP* library were used, where 5*s*^2^5*p*^6^6*s*^1^ and 4*s*^2^4*p*^5^ were treated as valence electrons for Cs and Br atoms, respectively. A unit cell was used for these calculations. The plane-wave cutoff energy was 800 eV and a 2 × 2 × 2 Monkhorst–Pack *k*-point mesh was applied. The convergence thresholds for the self-consistent field calculations and geometric optimizations were 1.0 × 10^−8^ eV and 1.0 × 10^−3^ eV Å^−1^, respectively.

## 3. Results and discussion

### 3.1. Simultaneous multiline detection using the TES in a single XANES scan

The energy resolution of the TES was compared with that of an SDD and a CAS based on the FWHM of the XES spectra using the Cs *L*β_1_ line (Fig. S1 in the supporting information). The FWHM values were ∼6.5, 130 and 3.8 eV for the TES, SDD and CAS, respectively. The energy resolution of the TES is much better than that of the SDD. In a comparison between the TES and the CAS, the two systems differ in both intensity and measurement time. The intensity of the XES spectrum using the TES is approximately one order of magnitude lower than that measured with the CAS, owing to the count rate limitation of the TES (∼1000 counts s^−1^). An XES spectrum acquired with the TES requires only about 5 s, whereas that measured with CAS takes approximately 8 min because the crystal must be realigned for each emission energy, with additional time required for crystal motion in addition to the dwell time of 1 s per point for more than 100 points. A quantitative comparison among the TES, SDD and CAS was also reported in our previous study (Takahashi *et al.*, 2025 ▸).

**Figure 2:**
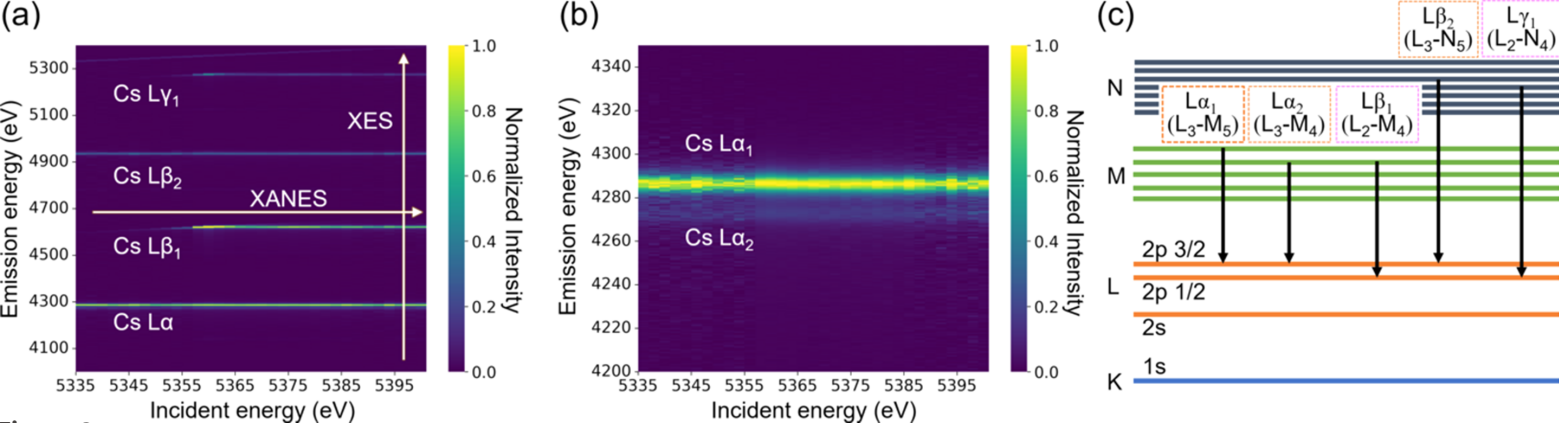
(*a*) 2D image of the intensity of emitted X-rays, normalized by the maximum intensity of La_1_, as a function of incident and emission X-ray energies for CsNO_3_. (*b*) Enlarged 2D map of part (*a*) to visualize the *L*α_1_ and *L*α_2_ features. (*c*) The transitions of the detected emission lines and their Siegbahn notations (*e.g.L*α_1_) and IUPAC notations (*e.g.L*_3_–*M*_5_).

The main advantage of TES is its ability to simultaneously measure emitted X-rays across a wide energy range with high energy resolution. Therefore, a XANES measurement – which involves scanning the incident energy over a defined range – produces a 2D map showing the intensity of the emission X-ray as a function of both incident and emission X-ray energies (Fig. 2 ▸ and Fig. S2 in the supporting information). When the incident energy was varied from 5335 to 5400 eV (the Cs *L*_II_-edge XANES region), the emission lines corresponding to Cs *L*α_1_, *L*α_2_ and *L*β_2_ were consistently observed. By contrast, the Cs *L*β_1_ and *L*γ_1_ lines emerged only after the scan began because the absorption energies of the Cs *L*_II_-edge and *L*_III_-edge are 5359 and 5012 eV, respectively. In this energy range, the I *L*-edges are also excited. As a result, the CsI spectrum exhibited the *L*β_1_ line (∼4221 eV) as a clear line and the *L*β_2_ (∼4506 eV) and *L*γ_1_ (∼4801 eV) lines as faint lines [Fig. S2(*a*)]. These lines are also visible in the 1D XES image of CsI [Fig. S2(*b*)]. A single TES measurement per­for­med during an incident energy scan enables the simultaneous acquisition of XANES spectra corresponding to multiple emission lines, as well as XES spectra over a wide range of incident X-ray energies. In addition, the high energy resolution of the TES enables it to resolve the *L*α_1_ (∼4285 eV) and *L*α_2_ (∼4272 eV) lines, as shown in Fig. 2 ▸(*b*).

### 3.2. HERFD-XANES spectra

**Figure 3:**
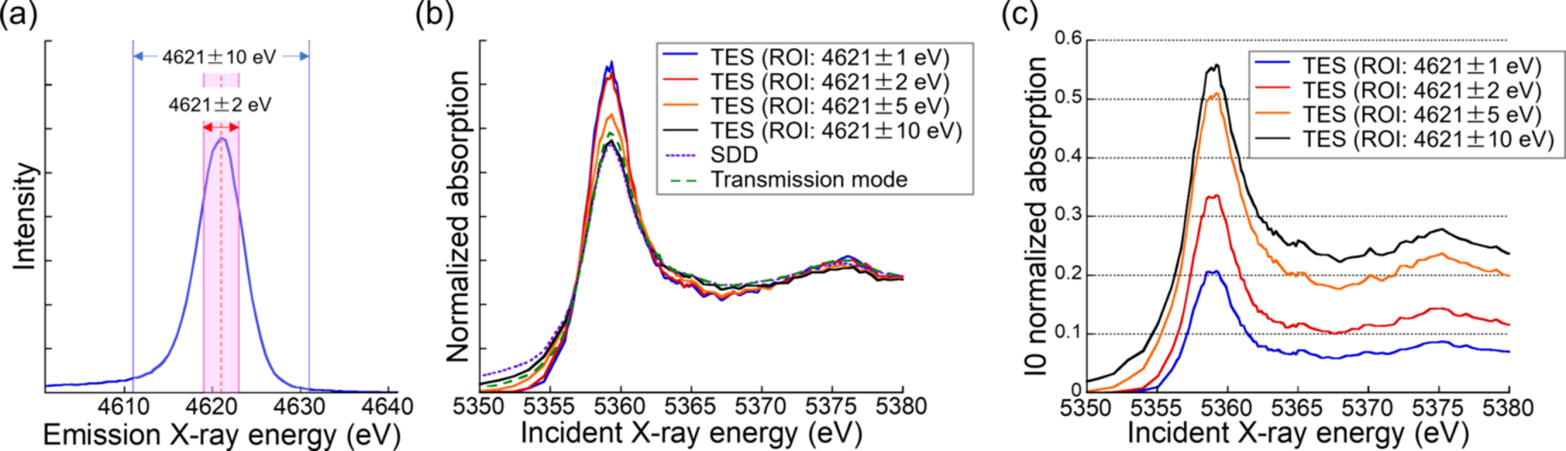
(*a*) RXES of CsNO_3_ at an incident X-ray energy of 5359 eV with selected ROI ranges. (*b*) Cs *L*_II_-edge XANES spectra of CsNO_3_ collected using the TES with various ROIs, compared with spectra obtained using the SDD and transmission mode. (*c*) XANES spectra collected with the TES using various ROIs, normalized only by the incident X-ray intensity (*I*_0_) and without post-edge normalization.

Fig. 3 ▸ shows Cs *L*_II_-edge XANES spectra of CsNO_3_ col­lected using the TES with various regions of interest (ROIs), alongside spectra obtained using the SDD and transmission mode. The white line appears sharper as the ROI range narrows, enhancing the ability to distinguish subtle differences between samples. The spectrum acquired *via* the TES with an ROI of 4621 ± 10 eV closely resembles those obtained using the SDD and transmission mode. However, when the XRF is confined to 4621 ± 5, 4621 ± 2 and 4621 ± 1, the XANES peak became much sharper owing to the suppression of lifetime broadening. This behaviour can be attributed to the energy resolution of the TES in this study (6.5 eV), being slightly larger than the natural linewidth of the Cs *L*β_1_ line (3–5 eV). The details of the mechanism behind the white-line sharpening are discussed elsewhere (Glatzel & Bergmann, 2005 ▸; Glatzel *et al.*, 2013 ▸). The narrower ROI decreases the efficiency of the XANES measurements, as shown in Fig. 3 ▸(*c*). The total count over the XANES region with an ROI of ±10 eV was 1.2 times higher than that with ±5 eV, 2.0 times higher than that with ±2 eV and 3.3 times higher than that with ±1.0 eV.

**Figure 4:**
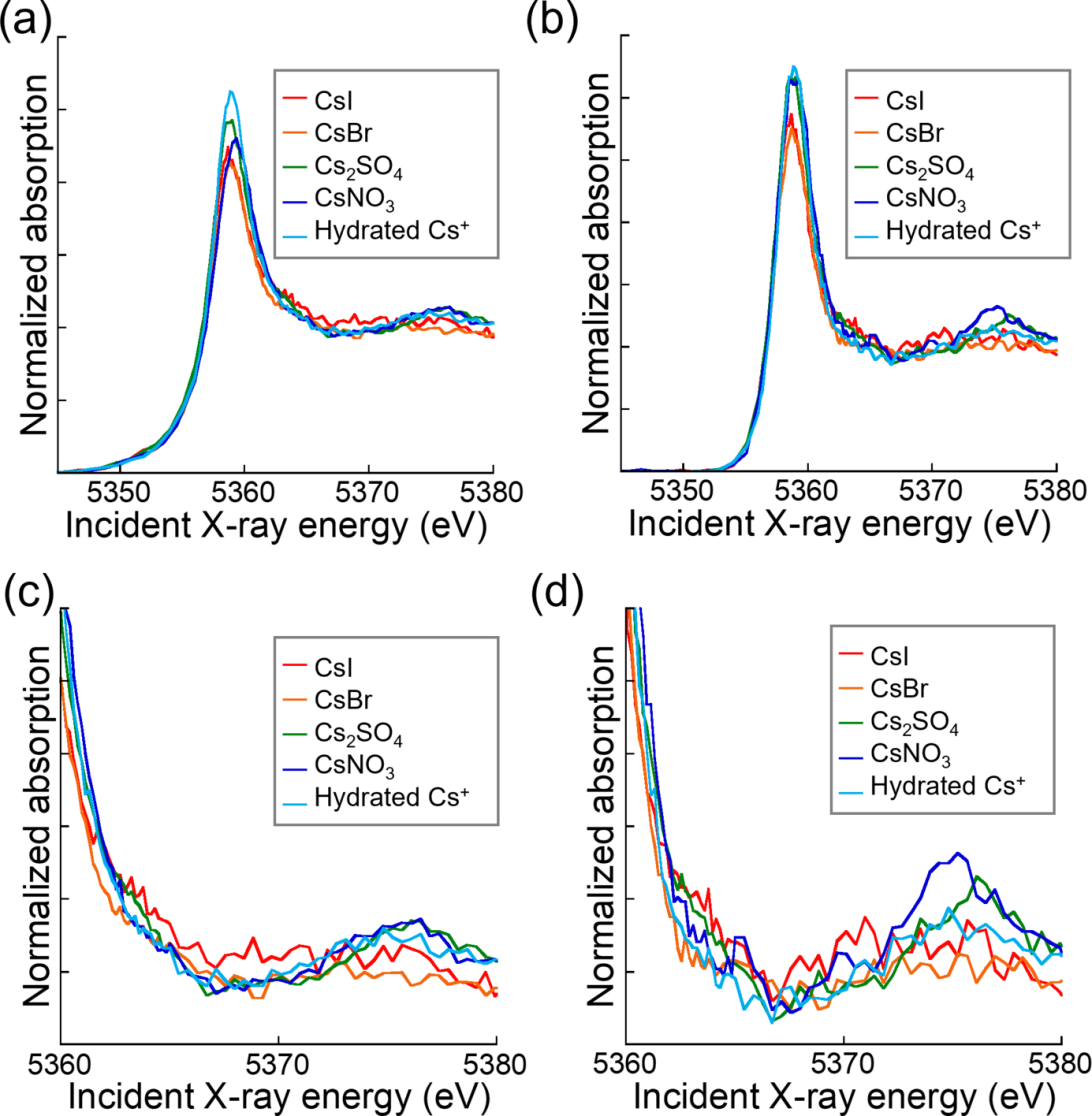
XANES spectra of various Cs compounds with ROIs at 4621 ± 10 eV and 4621 ± 1 eV: (*a*) ROI = 4621 ± 10 eV, (*b*) ROI = 4621 ± 1 eV, (*c*) ROI = 4621 ± 10 eV and (*d*) ROI = 4621 ± 1 eV. Part (*c*) is an enlarged view of part (*a*) and part (*d*) is an enlarged view of part (*b*).

Fig. 4 ▸ shows XANES spectra of various Cs compounds measured using the TES. When the ROI range is set to ±1 eV, the white-line peaks appear sharper and the post-edge structure (5360–5380 eV) exhibits slight variations depending on the compound. These spectra can be regarded as a type of HERFD-XANES. In the post-edge region, CsNO_3_, Cs_2_SO_4_ and hydrated Cs^+^ exhibit larger fluctuations, while CsI and CsBr show comparatively flatter spectra. These differences may be attributed to the crystal structures and bonding states. Fig. S3 shows their crystal structures, obtained by DFT calculations in a previous study (Yamaguchi *et al.*, 2025 ▸) and visualized using *VESTA* (Momma & Izumi, 2011 ▸). CsI and CsBr have a CsCl-type crystal structure, while CsNO_3_, Cs_2_SO_4_ and hydrated Cs^+^ possess a structure in which molecules are coordinated to Cs. Previous studies have reported that the former exhibit more covalent characters, while the latter are more ionic. These characteristics may account for the differences observed in the XANES spectra.

To the best of our knowledge, this is the first report of HERFD-XANES measurements per­for­med using the energy-dispersive detector without using a CAS for energies above 2 keV. Cs *L*_II_-edge XANES spectra can be obtained using either the *L*β_1_ or *L*γ_1_ emission lines. These two lines yield similar spectra (Fig. S4), which allow clearer distinction between samples in the spectra than those obtained by SDD, indicating that both are suitable for Cs *L*_II_-edge XANES analysis. *L*β_1_ is generally preferred owing to its higher intensity and greater separation from the incident X-ray energy, whereas *L*γ_1_ may be more appropriate where interfering elements are present. For example, in samples with high titanium (Ti) content, Ti *K*α fluorescence (4512 eV) can interfere with Cs *L*α_1_ (4285 eV) and *L*β_1_ (4618 eV). By contrast, Cs *L*γ_1_ (5279 eV) is well separated from Ti *K*α, making it a more effective choice in such cases. This is often the case in environmental chemistry because the abundance of Ti in the bulk silicate Earth is far larger than that of Cs; their concentrations are 1230 and 0.021 ppm, respectively (Fischer & McDonough, 2025 ▸).

Moreover, the ability of the TES to detect multiple XRF lines may provide a significant advantage for obtaining HERFD-XANES. The sharpening of the XANES spectra is determined by the lifetime broadening of the electron energy levels responsible for each XRF – for example, 3*d*_3/2_ and 4*d*_3/2_ electrons in the case of *L*β_1_ and *L*γ_1_, respectively. By selecting transitions associated with electrons that have much smaller lifetime broadening, it is possible to achieve sharper HERFD-XANES spectra. However, this improvement was not apparent in the data shown in Fig. S4, possibly due to the energy resolution of the TES.

**Figure 5:**
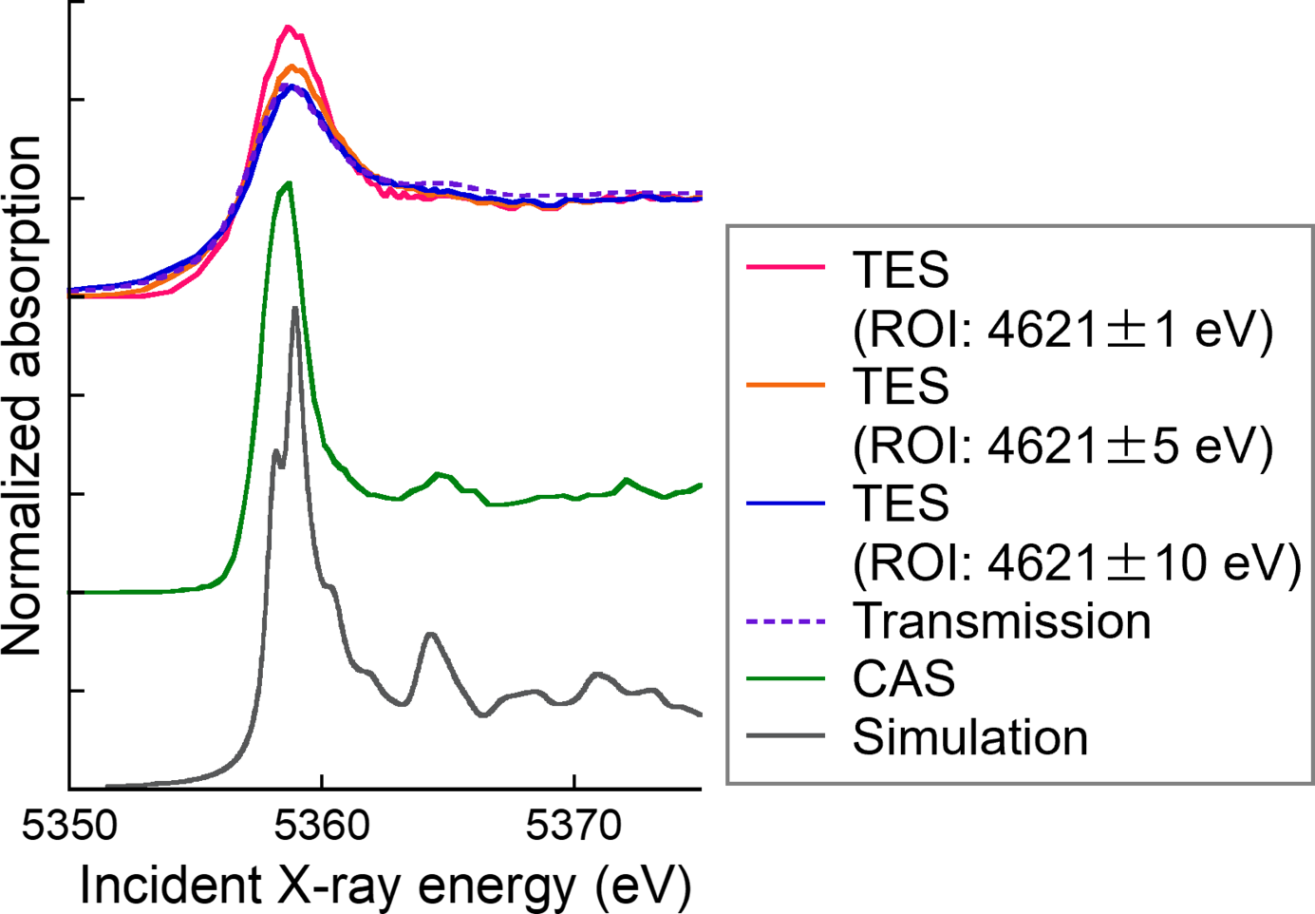
XANES spectra of CsBr measured using the TES with various ROIs, compared with spectra obtained by transmission mode and CAS, and with DFT-simulated spectra.

The spectra of CsBr collected using the TES were compared with those obtained using transmission mode, a CAS and DFT simulations (Fig. 5 ▸). The spectrum collected using a CAS exhibits a very sharp white line and a secondary peak at ∼5365 eV. These characteristics are also present in the simulated spectrum, providing theoretical support for the CAS measurements. The spectrum obtained *via* a CAS was collected using a fixed fluorescence energy with an ROI range of <0.4 eV. Although the TES also provides high-energy resolution, its count rate is limited, and a minimum practical ROI range of 1 eV is required to maintain a good signal-to-noise ratio. These findings suggest that obtaining HERFD-XANES spectra with sharp features requires further improvements in both the energy resolution and the maximum count rate of the TES. The energy resolution of the TES can be improved by reducing the size of individual sensor elements or by employing more advanced sensor materials. By contrast, increasing the count rate requires expanding the effective area by increasing the number of pixels and reducing heat generation in the system (Yamada *et al.*, 2021 ▸).

In our previous study, HERFD-XANES spectra of several Cs compounds were measured using a CAS and decomposed using Gaussian and arctangent functions (Yamaguchi *et al.*, 2025 ▸). The centroid energies of their white lines showed a strong correlation with the nucleophilic constant (*E_n_*), which quantifies the covalency of ligands, consistent with HSAB theory (Edwards, 1954 ▸). These results demonstrated that shifts in the white-line energy of HERFD-XANES spectra can be used to evaluate covalency, with a shift to lower energy generally indicating greater covalency. This conclusion is further supported by DFT calculations of Cs–ligand distances and crystal orbital Hamilton population chemical bonding analysis, both of which yielded consistent trends. Generally, the peak shift of XANES spectra is sensitive to the electronic state of the absorbing atom. A change in the oxidation state of the absorbing atom produces a larger shift and that in its local coordination environment produces a smaller shift. Since Cs exists only in the monovalent state, the peak shift of Cs XANES spectra reflects the change in the local coordination environment. In this study, the HERFD-XANES spectra obtained by the TES were also decomposed and their centroid energies were determined [Fig. S5(*a*)]. Although the centroid energies showed a good correlation with the *E_n_* [Fig. S5(*b*)], the correlation coefficient *R* value (0.87) was lower than that obtained from the CAS measurements (0.97) in our previous study (Yamaguchi *et al.*, 2025 ▸), possibly due to the lower energy resolution of the TES.

Although the energy resolution and count rate is lower than the CAS, the TES offers several advantages. An advantage of HERFD-XANES measured by the TES is that the ROI is easily changed after the measurement. A narrow ROI brings a spectrum with shaper peaks, but it decreases the efficiency [Fig. 3 ▸(*c*)]. Therefore, a wider ROI can be better for a dilute sample. This flexibility of ROI is particularly effective for natural samples because they often include a low concentration of a target element and high concentrations of matrix elements. In addition, although the HERFD-XANES spectra obtained by *L*β_1_ and *L*γ_1_ had similar features in this study, HERFD-XANES spectra measured at different fluorescence emission lines can in general exhibit different spectral shapes (Glatzel *et al.*, 2013 ▸). Thus, a simultaneous measurement using multiple lines is also valuable.

As a practical example, the TES is effective for a sample containing multiple elements and interference by a matrix element. Our previous study employed the TES to investigate speciation of Cs in a radiocesium-bearing microparticle (CsMP) emitted from the Fukushima Dai-ichi Nuclear Power Plant accident in Japan. The CsMP has a high concentration of Ti, causing a severe interference when the fluorescence X-rays were detected by SDD. However, the TES successfully distinguished the Cs *L*α_1_ (∼4285 eV) and Ti *K*α_1_ (∼4512 eV) lines, and produced Cs *L*_III_-edge XANES spectra.

### 3.3. RXES spectra

This section is focused on RXES spectra. RXES spectra were collected at incident X-ray energies of 5359, 5370, 5380 and 5390 eV, with 5359 eV being just above the Cs *L*_II_-edge absorption edge. The RXES spectra of various Cs compounds exhibited similar overall features (Fig. S6).

As discussed in the previous section, the white-line energy of HERFD-XANES spectra can be used as an indicator of bonding state. Because RXES also reflects the excitations of inner electrons, it similarly provides insights into covalency. Herein, the relative intensities of emission lines were examined, a concept previously explored for elements such as copper, barium, lanthanum and cerium (Kawai *et al.*, 1993 ▸; Baydaş *et al.*, 1998 ▸).

The *L*α_1_ and *L*β_1_ peaks in the RXES spectra, normalized by the maximum intensity of the *L*α_1_, were fitted using the Voigt function, which is a convolution of the Gaussian and Lorentzian functions. The fitting was per­for­med using the following equation:
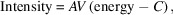
where *A* is the amplitude, *C* is the centroid energy and *V* is the Voigt function. The Voigt function includes two parameters: σ, the standard deviation of the Gaussian component, and γ, the half width at half maximum (HWHM) of the Lorentzian component. Thus, the fitting involved four variables: *A*, *C*, σ and γ. The fitting results are shown in Fig. S7 and summarized in Table S1.

**Figure 6:**
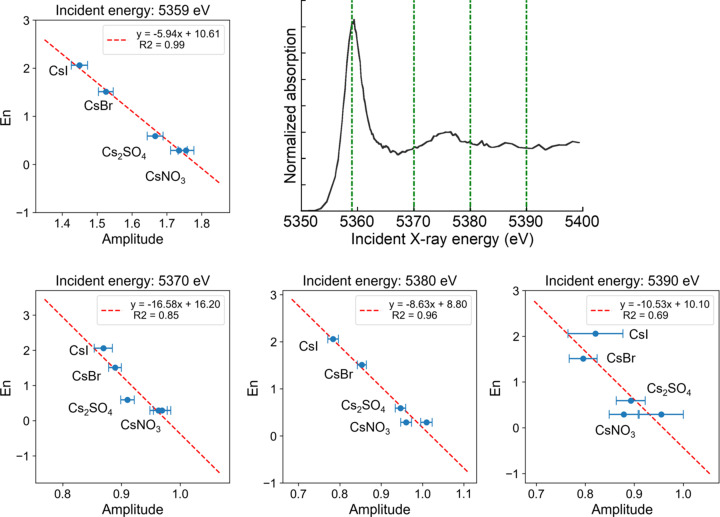
Normalized *L*β_1_ amplitudes (divided by *L*α_1_) plotted against *E_n_* for various incident energies and a typical Cs *L*_II_-edge XANES spectrum to indicate the incident energies.

Fig. 6 ▸ plots the normalized amplitude, defined as the amplitude of *L*β_1_ divided by that of *L*α_1_, plotted against the *E_n_* values. A strong correlation was observed regardless of the incident energy: samples with lower normalized amplitudes exhibited higher *E_n_* values, indicating greater covalency. The large error bars observed at an incident energy of 5390 eV are attributable to weak emission line intensities at this energy.

This analysis was also applied to the *L*γ_1_ line (Fig. S8). However, the correlations with *E_n_* were much weaker than those observed for *L*β_1_. This is likely because the energy of the Cs *L*_III_ absorption edge (5012 eV) lies below the energy of the *L*γ_1_ emission line (5279 eV), resulting in partial absorption of the *L*γ_1_ signal. These findings suggest that the normalized emission line amplitude can serve as an indicator of covalency if absorption effects from included elements and the incident X-ray energy are carefully considered.

### 3.4. RIXS planes

**Figure 7:**
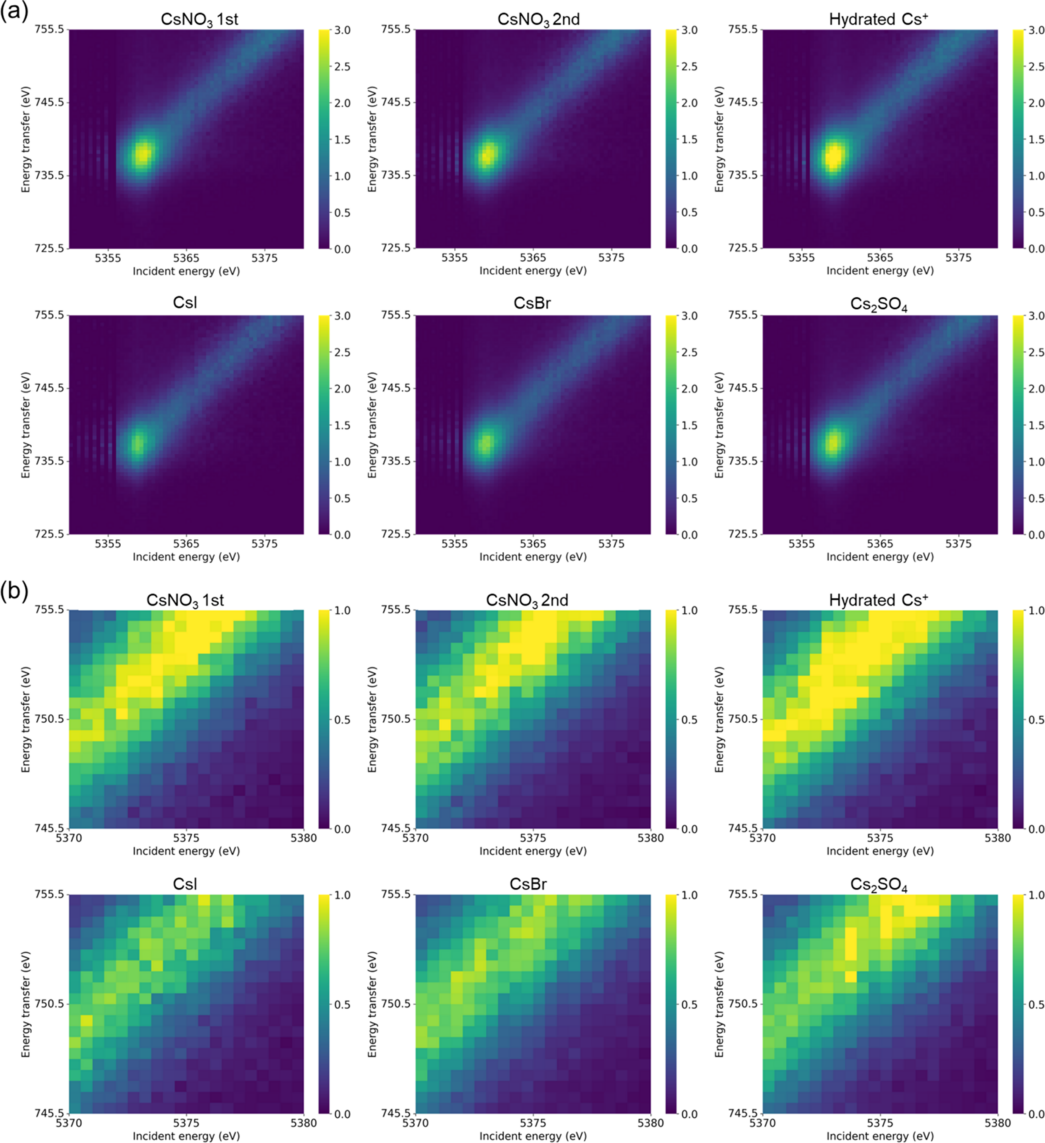
(*a*) RIXS planes according to *L*β_1_ and (*b*) their enlarged images. The energy transfer was obtained by subtracting the emission energy from the incident energy. The colour bar shows intensity normalized by the maximum intensity of *L*α_1_.

In addition, RIXS planes were plotted for *L*β_1_ and *L*γ_1_ (Fig. 7 ▸ and Fig. S9). RIXS, which combines X-ray absorption and emission, has recently attracted attention as a strong tool for probing fine electronic energy levels, mainly in the field of materials chemistry (de Groot *et al.*, 2024 ▸). Here, the RIXS planes was created based on the data used to generate 2D maps of emitted X-ray intensity (Fig. 2 ▸) and the 1D XES spectra (Fig. S6). The difference between Figs. 2 ▸ and 7 ▸ lies in the *y* axis: while the *y* axis in Fig. 2 ▸ represents the emission X-ray energy, that in Fig. 7 ▸ represents the energy transfer, which is defined as the difference between the incident and emission energies. The energy step size is 0.5 eV for both the incident energy and the energy transfer axes. In the pre-edge region of the Cs *L*_II_-edge XANES, *i.e.* below 5355 eV, the energy step size of the incident X-ray increases to about 1.0 eV. Consequently, the intensities at some data points in this region were plotted as zero.

A single scan with the TES simultaneously provided two RIXS planes corresponding to *L*β_1_ and *L*γ_1_. The RIXS planes for *L*β_1_ normalized by the maximum intensity of *L*α_1_ exhibited different intensities depending on the Cs compounds [Fig. 7 ▸(*a*)]. For example, the maximum intensities of CsI and CsBr were smaller than those of the other compounds, consistent with the RXES analysis (Fig. 6 ▸). In addition, magnified views of the post-edge region showed different features depending on the compounds [Fig. 7 ▸(*b*)], in agreement with the HERFD-XANES results (Fig. 4 ▸). These trends were also observed in the RIXS planes for *L*γ_1_ (Fig. S9). However, the differences among the samples were less pronounced, likely due to the lower signal intensity. Furthermore, in the RIXS planes of *L*γ_1_, hydrated Cs^+^ showed a particularly strong intensity. A possible explanation is that hydrated Cs^+^ is in the liquid state, resulting in a different absorption effect of *L*γ_1_ at the Cs *L*_III_-edge compared with the other solid samples. These results suggest that the RIXS planes obtained with the TES can distinguish different chemical species.

In addition, the simultaneous measurement with the TES has the potential to reveal unexpected electronic transitions. As discussed in the *Introduction*, a previous report demonstrated the potential of the TES for off-resonant XES (Uhlig *et al.*, 2015 ▸), and the present study represents, to the best of our knowledge, the first observation of RIXS using an energy-dispersive detector operating in the energy range above 2 keV. Although the current energy resolution is insufficient to resolve energy losses associated with phonons (100 meV or less) or ligand-field excitations (several eV), which are often considered in RIXS studies (de Groot *et al.*, 2024 ▸), future improvements in the energy resolution and count rate of the TES are expected to enable RIXS and HERFD-XANES analyses comparable with those achieved with CAS.

### 3.5. Potential of the TES

The results presented above demonstrate that the TES offers several practical and methodological advantages, even though its energy resolution is lower than that of the CAS. The HERFD-XANES spectra measured with the TES reveal more detailed features than those obtained with an SDD, enabling discrimination of chemical species and bonding states.

A key advantage of the TES over the CAS is the simultaneous collection of multiple fluorescence lines, such as *L*β_1_ and *L*γ_1_, together with the flexibility in defining ROI in energy. The RXES spectra obtained with the TES can also distinguish chemical species; in particular, the *L*β_1_ intensity normalized by *L*α_1_ serves as a sensitive indicator of the bonding state. Importantly, the acquisition of both *L*β_1_- and *L*α_1_-resolved RXES spectra required for this analysis would necessitate separate measurements and repeated realignment of the analyzing crystals when using a CAS, resulting in substantially longer acquisition times. In contrast, the TES can obtain these datasets simultaneously in a single scan. Furthermore, the RIXS planes measured with the TES provide additional information for distinguishing chemical species. The ability to easily acquire RIXS data is particularly valuable for HERFD-XANES measurements, as RIXS plays a crucial role in the proper interpretation of HERFD-XANES spectra (Glatzel & Bergmann, 2005 ▸).

In summary, the advantages of the TES over the CAS are as follows: (i) the TES is easier to install, as no alignment of analyzing crystals is required; (ii) the TES can simultaneously collect X-ray fluorescence signals over a wide energy range (>∼2 keV), enabling rapid measurements and the simultaneous detection of HERFD at multiple emission lines; (iii) no crystal exchange is required, reducing experimental setup effort and time; (iv) the TES is applicable in the tender X-ray range, where CAS-based approaches are generally less suitable (Uhlig *et al.*, 2015 ▸); and (v) the TES can achieve a high effective detection efficiency by being placed close to the sample, although in the present study this advantage is partly limited by the count-rate capability, which is expected to improve in future developments.

Although the energy resolution of the TES was inferior to that of the CAS in this study, TES performance continues to improve rapidly (*e.g.* Gottardi & Smith, 2023 ▸). For example, recent TES devices have achieved energy resolutions of 0.7 eV (FWHM) for Al *K*α (∼1487 eV) and 1.27 eV for Mn *K*α (∼5900 eV). In addition, the flexibility of TES design represents an important advantage. The energy resolution of a TES is approximately given by
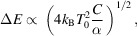
where *k*_B_, *T*_0_, *C* and α denote the Boltzmann constant, operating temperature, detector heat capacity and temperature sensitivity of the superconducting transition, respectively. Because these parameters depend on detector design and materials, TES performance can be tailored to specific experimental requirements.

Overall, this study highlights the unique capability of TES to perform simultaneous HERFD-XANES, RXES and RIXS analyses with energy-dispersive detection in the hard X-ray range.

## 4. Conclusion

This study analyzed several Cs compounds using the TES, which can measure XRF over a wide energy range with high resolution. By scanning the incident X-ray energy across the Cs *L*_II_-edge, the TES enabled the simultaneous acquisition of HERFD-XANES spectra, RXES spectra and RIXS planes for multiple emission lines in a single measurement. The energy resolution of the TES was significantly higher than that of conventional detectors such as SDD and SSD, allowing for the clear observation of fine spectral features.

HERFD-XANES spectra obtained with narrow ROI settings revealed subtle differences in the white line and post-edge structures among various Cs compounds. To the best of our knowledge, this is the first report of HERFD-XANES measurements per­for­med using an energy-dispersive detector operating in the energy range above 2 keV. RXES spectra were further analyzed by fitting the *L*α_1_ and *L*β_1_ emission lines using Voigt functions. The normalized amplitude of *L*β_1_ exhibited a strong correlation with the *E_n_*, indicating that RXES can serve as a useful probe for evaluating covalency. In addition, RIXS planes corresponding to *L*β_1_ and *L*γ_1_ emission lines were successfully acquired in a single scan. These simultaneous measurements provide valuable insights into the electronic structure of the target element and may reveal unexpected electronic transitions.

## Supplementary Material

Table S1 and Figures S1 to S9. DOI: 10.1107/S1600577526001682/ok5154sup1.pdf

## Data Availability

Additional data supporting the findings of this study are available from the corresponding author upon reasonable request.
