# Supplementary material for: Simultaneous measurements of HERFD-XANES, RXES and RIXS of caesium using a transition-edge sensor
Source: J Synchrotron Radiat. 2026 Apr 3;33(Pt 3):683–92. doi: 10.1107/S1600577526001682 (PMC13148627; doi:10.1107/S1600577526001682)
Supplement: Supplementary file 1 [file s-33-00683-sup1.pdf]

JOURNAL OF SYNCHROTRON RADIATION

Volume 33 (2026)

Supporting information for article:

Simultaneous measurements of HERFD-XANES, RXES, and RIXS of caesium using a transition-edge sensor

Akiko Yamaguchi, Shinya Yamada, Tadashi Hashimoto, Takuma Okumura, Yasuo Takeichi, Masahiko Okumura and Yoshio Takahashi

Table S1 Fitting results of emission lines of some Cs compounds. A, C, σ, γ, and R^2 represent the amplitude, the centroid energy, the standard deviation of the Gaussian component, the half-width at half-maximum of the Lorentzian component, and the coefficient of determination, respectively.

Emission line: Lα_1

Incident X-ray energy (eV)	Sample	*A*	error	*C*	error	σ	error	γ	error	R^2
5359	Cs_2SO_4	9.83	0.07	4286.35	0.01	1.98	0.04	2.22	0.06	1.00
	CsBr	9.94	0.07	4286.35	0.01	1.99	0.04	2.22	0.05	1.00
	CsI	9.74	0.10	4286.31	0.01	1.90	0.07	2.26	0.08	1.00
	$CsNO_3$	9.88	0.06	4286.37	0.01	1.96	0.04	2.21	0.05	1.00
	$CsNO_3$ 2nd	9.70	0.07	4286.33	0.01	2.00	0.05	2.10	0.06	1.00
	Hydrated Cs^+	9.85	0.07	4286.37	0.01	2.03	0.04	2.19	0.05	1.00
5370	Cs_2SO_4	9.49	0.09	4286.01	0.01	1.93	0.06	2.13	0.07	1.00
	CsBr	9.61	0.09	4286.06	0.02	2.14	0.06	2.01	0.08	1.00
	CsI	9.52	0.14	4286.15	0.02	1.91	0.10	2.12	0.12	1.00
	$CsNO_3$	9.34	0.11	4286.02	0.02	2.01	0.07	2.09	0.09	1.00
	$CsNO_3$ 2nd	9.22	0.11	4286.12	0.02	1.94	0.08	2.04	0.10	1.00
	Hydrated Cs^+	9.52	0.11	4286.04	0.02	1.82	0.08	2.21	0.09	1.00
5380	Cs_2SO_4	9.53	0.10	4285.98	0.02	2.10	0.06	1.96	0.08	1.00
	CsBr	9.54	0.09	4285.97	0.01	2.01	0.06	2.07	0.08	1.00
	CsI	9.51	0.13	4285.95	0.02	1.88	0.09	2.16	0.11	1.00
	$CsNO_3$	9.40	0.10	4286.00	0.02	2.06	0.07	1.92	0.09	1.00
	$CsNO_3$ 2nd	9.32	0.10	4286.00	0.02	1.94	0.06	2.01	0.08	1.00
	Hydrated Cs^+	9.41	0.13	4286.00	0.02	2.13	0.08	1.93	0.11	1.00
5390	Cs_2SO_4	9.58	0.26	4285.87	0.04	2.25	0.16	1.81	0.22	0.99
	CsBr	8.92	0.29	4285.84	0.05	1.83	0.19	2.14	0.24	0.99
	CsI	8.67	0.57	4286.02	0.08	1.93	0.33	2.17	0.44	0.97
	$CsNO_3$	8.17	0.25	4286.04	0.05	1.48	0.22	2.37	0.23	0.99
	$CsNO_3$ 2nd	8.21	0.34	4286.00	0.05	2.12	0.21	1.58	0.30	0.99
	Hydrated Cs^+	8.89	0.34	4285.89	0.06	1.72	0.25	2.20	0.29	0.98

(Continued)

Emission line: Lα_2

Incident X-ray energy (eV)	Sample	*A*	error	*C*	error	σ	error	γ	error	R^2
5359	Cs_2SO_4	2.05	0.08	4272.31	0.09	0.00	278.00	4.62	0.40	1.00
	CsBr	2.12	0.09	4272.28	0.09	0.00	414.67	4.78	0.40	1.00
	CsI	2.27	0.10	4272.08	0.15	0.00	373.64	5.37	0.28	1.00
	$CsNO_3$	2.10	0.07	4272.34	0.09	0.00	535.57	4.75	0.21	1.00
	$CsNO_3$ 2nd	2.05	0.08	4272.29	0.10	0.00	439.02	4.58	0.30	1.00
	Hydrated Cs^+	2.17	0.08	4272.44	0.09	0.00	331.19	4.97	0.37	1.00
5370	Cs_2SO_4	1.96	0.08	4272.00	0.13	0.00	452.25	4.54	0.29	1.00
	CsBr	2.13	0.09	4271.99	0.13	0.00	0.42	4.68	0.22	1.00
	CsI	2.31	0.14	4271.88	0.21	0.00	19.33	5.12	0.35	1.00
	$CsNO_3$	1.93	0.14	4271.93	0.15	0.00	379.24	4.59	0.72	1.00
	$CsNO_3$ 2nd	1.99	0.10	4271.94	0.16	0.00	58.70	4.58	0.27	1.00
	Hydrated Cs^+	2.04	0.10	4271.91	0.17	0.00	242.53	4.75	0.28	1.00
5380	Cs_2SO_4	2.18	0.09	4271.98	0.14	0.00	214.76	4.90	0.24	1.00
	CsBr	2.14	0.09	4272.17	0.13	0.00	451.57	4.80	0.28	1.00
	CsI	2.15	0.12	4271.73	0.19	0.00	33.81	4.85	0.31	1.00
	$CsNO_3$	2.24	0.10	4272.18	0.15	0.00	322.11	5.05	0.26	1.00
	$CsNO_3$ 2nd	2.14	0.10	4272.25	0.14	0.00	759.86	4.78	0.28	1.00
	Hydrated Cs^+	2.00	0.16	4272.14	0.17	0.55	2.49	4.46	0.80	1.00
5390	Cs_2SO_4	2.12	0.26	4272.52	0.40	0.00	16.44	5.06	0.69	0.99
	CsBr	1.75	0.36	4272.11	0.39	1.25	2.64	3.83	1.94	0.99
	CsI	2.40	0.88	4271.91	0.86	0.00	2323.69	5.96	4.43	0.97
	$CsNO_3$	1.81	0.24	4271.56	0.46	0.00	772.41	4.90	0.78	0.99
	$CsNO_3$ 2nd	2.18	0.42	4272.40	0.46	0.00	2170.81	4.78	1.80	0.99
	Hydrated Cs^+	1.83	0.36	4271.68	0.71	0.00	2114.68	5.42	1.25	0.98

(Continued)

Emission line: Lβ_1

Incident X-ray energy (eV)	Sample	*A*	error	*C*	error	σ	error	γ	error	R^2
5359	Cs_2SO_4	16.38	0.19	4620.95	0.02	1.98	0.07	1.23	0.09	1.00
	CsBr	15.16	0.18	4620.96	0.02	1.95	0.07	1.31	0.10	1.00
	CsI	14.12	0.17	4620.93	0.02	1.97	0.08	1.25	0.10	1.00
	$CsNO_3$	17.34	0.20	4620.79	0.02	1.97	0.07	1.21	0.09	1.00
	$CsNO_3$ 2nd	16.82	0.19	4620.85	0.02	1.94	0.07	1.15	0.09	1.00
	Hydrated Cs^+	19.85	0.23	4620.94	0.02	2.00	0.07	1.25	0.09	1.00
5370	Cs_2SO_4	8.64	0.07	4620.70	0.02	1.43	0.10	2.86	0.08	1.00
	CsBr	8.54	0.06	4620.70	0.02	1.56	0.08	2.72	0.07	1.00
	CsI	8.28	0.08	4620.81	0.02	1.48	0.10	2.61	0.09	1.00
	$CsNO_3$	9.05	0.08	4620.61	0.02	1.44	0.11	2.85	0.09	1.00
	$CsNO_3$ 2nd	8.88	0.08	4620.64	0.02	1.29	0.11	2.80	0.09	1.00
	Hydrated Cs^+	10.35	0.10	4620.68	0.02	1.45	0.11	2.72	0.09	1.00
5380	Cs_2SO_4	9.02	0.08	4620.85	0.02	1.79	0.08	2.35	0.08	1.00
	CsBr	8.14	0.07	4620.81	0.02	1.62	0.08	2.54	0.08	1.00
	CsI	7.46	0.07	4620.84	0.02	1.72	0.10	2.52	0.09	1.00
	$CsNO_3$	9.49	0.08	4620.85	0.02	1.63	0.08	2.50	0.08	1.00
	$CsNO_3$ 2nd	8.95	0.08	4620.89	0.02	1.59	0.08	2.44	0.08	1.00
	Hydrated Cs^+	10.37	0.09	4620.84	0.02	1.62	0.08	2.46	0.08	1.00
5390	Cs_2SO_4	8.56	0.16	4620.60	0.05	1.21	0.21	2.72	0.16	0.99
	CsBr	7.10	0.10	4620.60	0.04	1.52	0.15	2.48	0.13	0.99
	CsI	7.12	0.12	4620.83	0.04	1.48	0.17	2.42	0.16	0.99
	$CsNO_3$	7.18	0.11	4620.63	0.04	1.63	0.16	2.65	0.14	0.99
	$CsNO_3$ 2nd	7.84	0.17	4620.77	0.05	1.33	0.25	2.68	0.20	0.98
	Hydrated Cs^+	8.81	0.18	4620.67	0.05	1.82	0.19	2.31	0.20	0.98

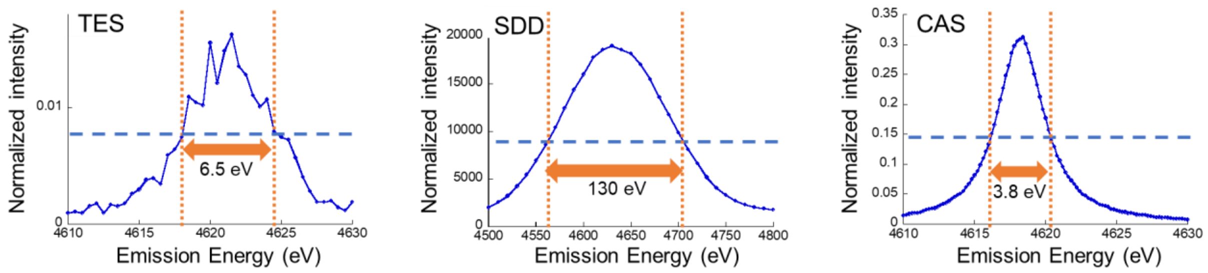

Figure S1 XES spectra of the Cs Lβ_1 line normalized by the I_0 intensity, and the energy resolutions of the TES, SDD, and CAS. The incident X-ray energies were 5.40 keV for the TES and SDD, and 5.45 keV for CAS.

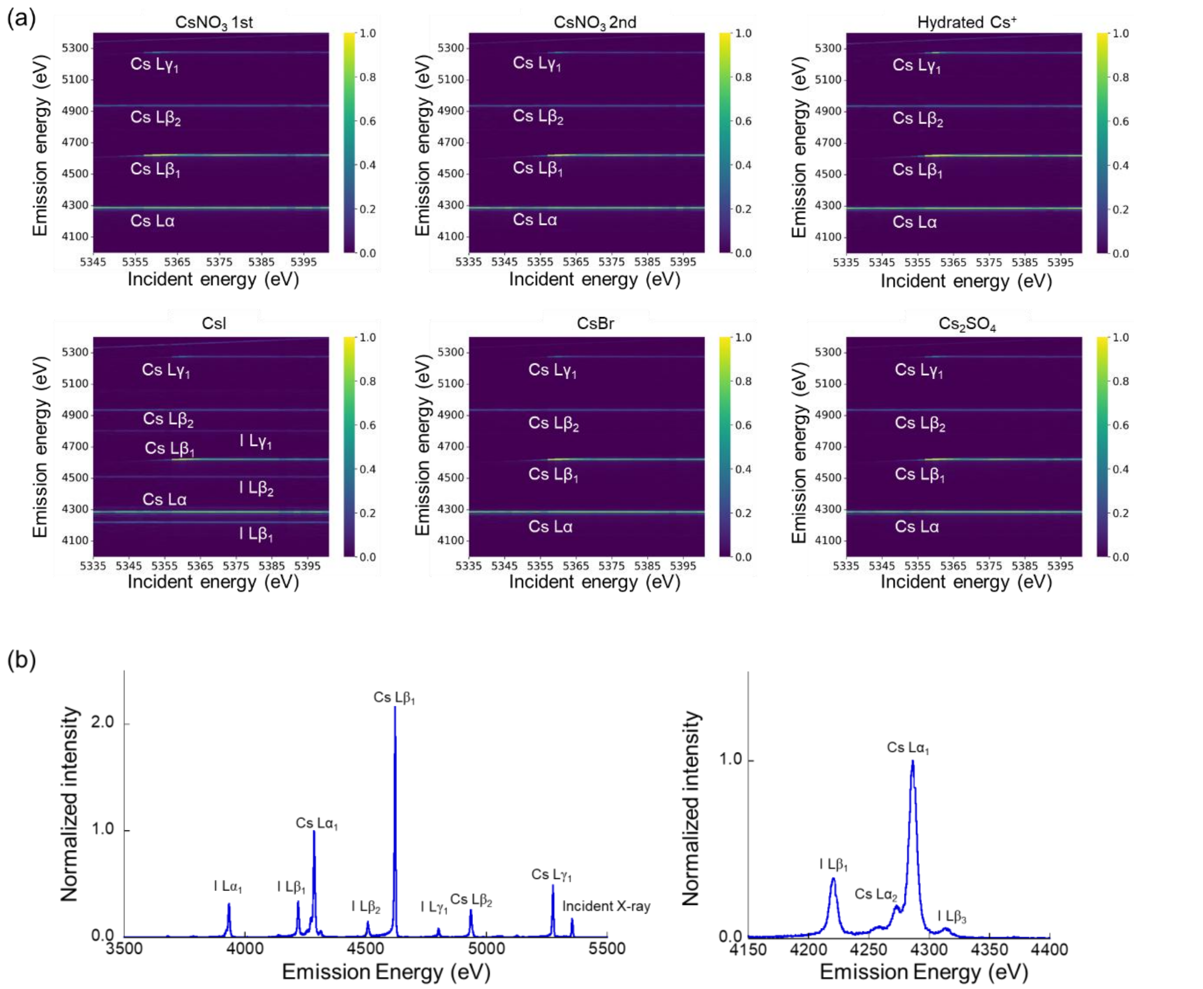

Figure S2 2D image of the intensity of emitted X-rays, normalized by Lα_1 intensity, as a function of incident and emission X-ray energies. The color bar shows normalized intensity. (b) 1D XES image of CsI when the incident energy is 5359 eV.

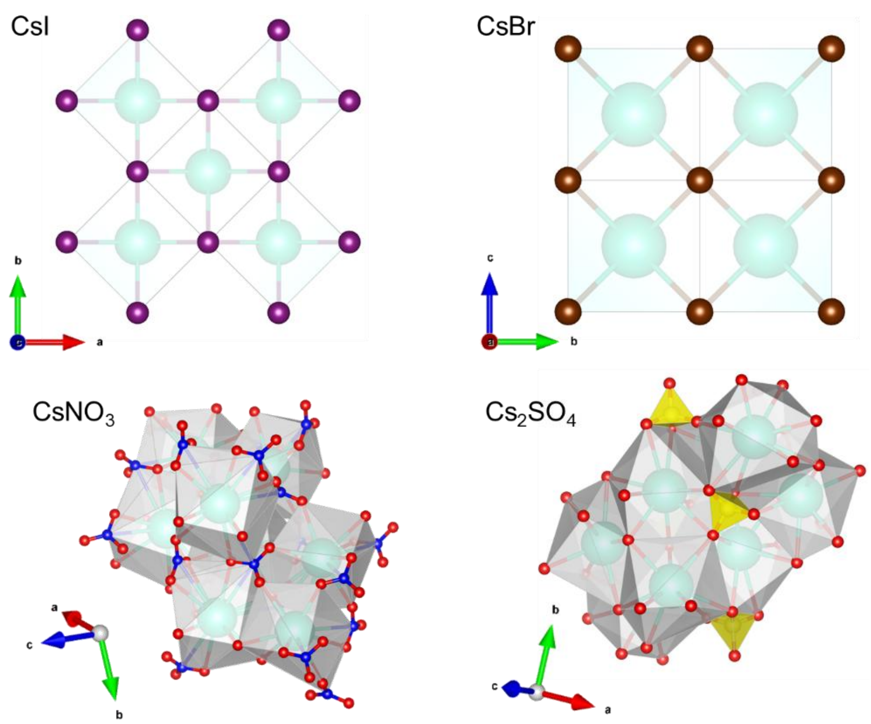

Figure S3 Crystal structures of Cs compounds. The light blue, purple, brown, red, blue, yellow show Cs, I, Br, O, N, and S, respectively.

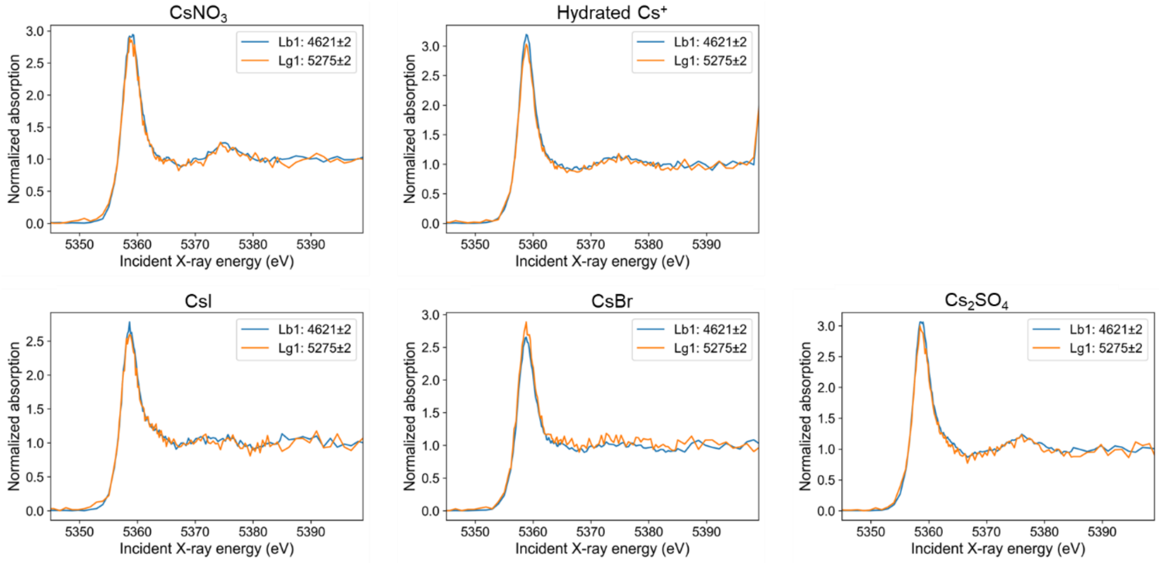

Figure S4 Cs L_{II}-edge spectra collected by using $L\beta_1$ and $L\gamma_1$.

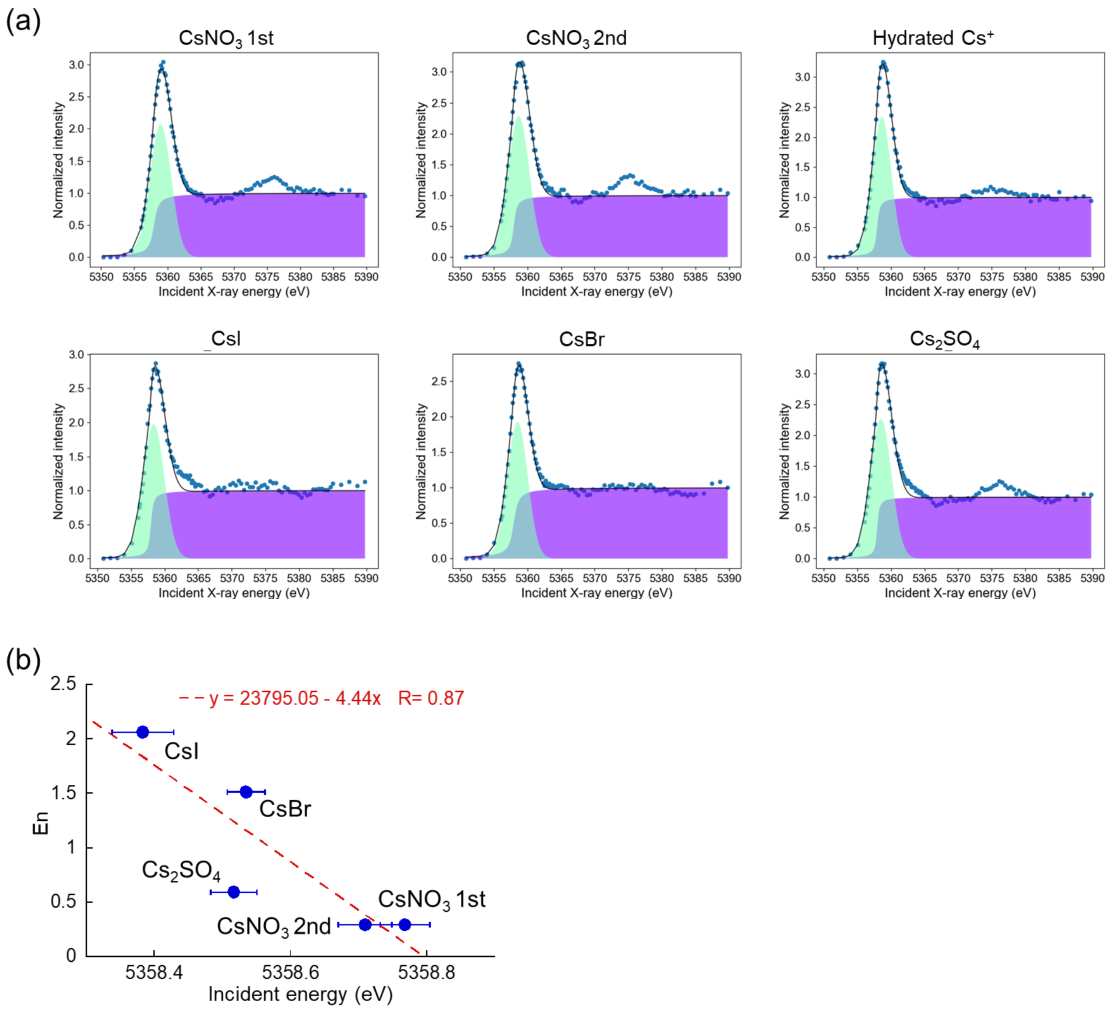

Figure S5 (a) Fitting results of XANES when theROI is 4621±1 eV. The blue dots and black line represent the measured data points and the total fitting result, respectively, while the green and purple areas show the fitting results obtained with gaussian and arctangent functions, respectively. (b) Centroid energies of the gaussian function fitted to the white line and En values. It should be noted that the incident energy of $CsNO_3$ in the first scan was calibrated using the XANES spectra collected with the SDD. A difference of 0.166 eV was observed between the first and second scans, possibly due to heating of the monochromator crystals.

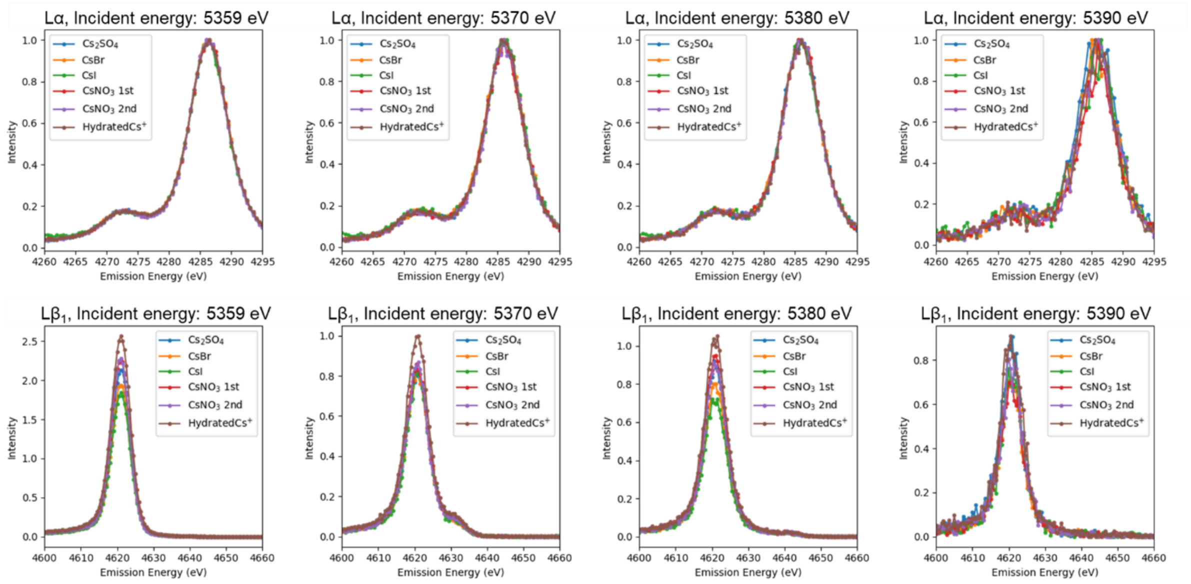

Figure S6 Cs RXES spectra for $L\alpha_1$, $L\alpha_2$ and $L\beta_1$, normalized by the maximum intensity of the $L\alpha_1$.

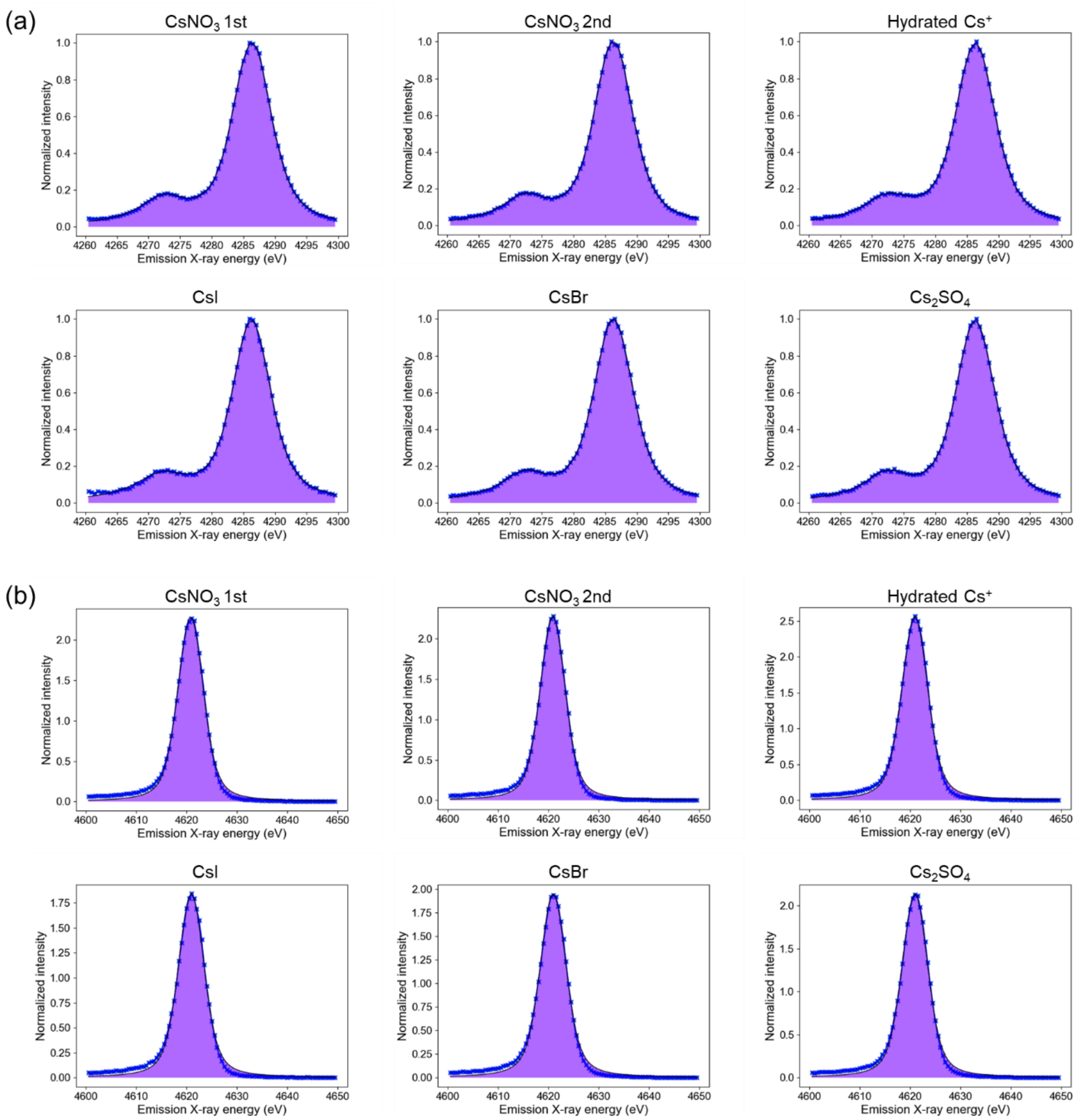

Figure S7 Fitting results of XES when the incident X-ray energy is 5359 eV for (a) Lα_1 and Lα_2, and (b) Lβ_1. Blue mark and purple area show the measured and fitted results, respectively.

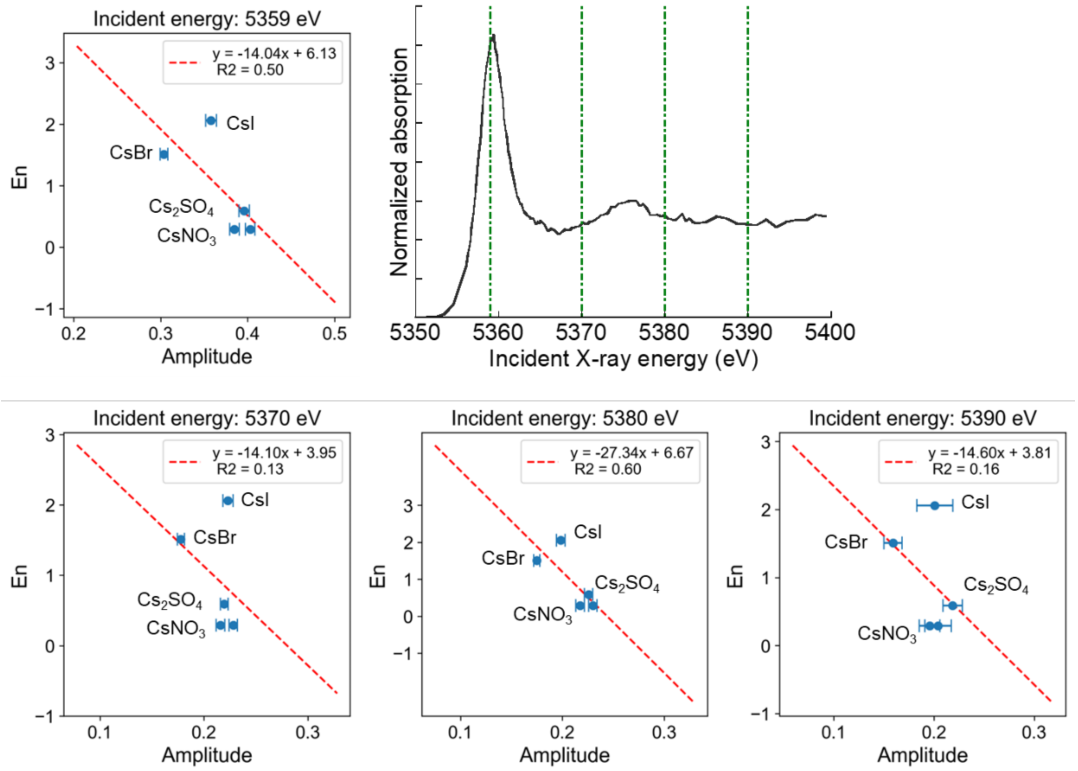

Figure S8 En values according to normalized amplitude Lγ_1 by Lα_1. The broken line shows the regression line and R2 means its coefficient of determination.

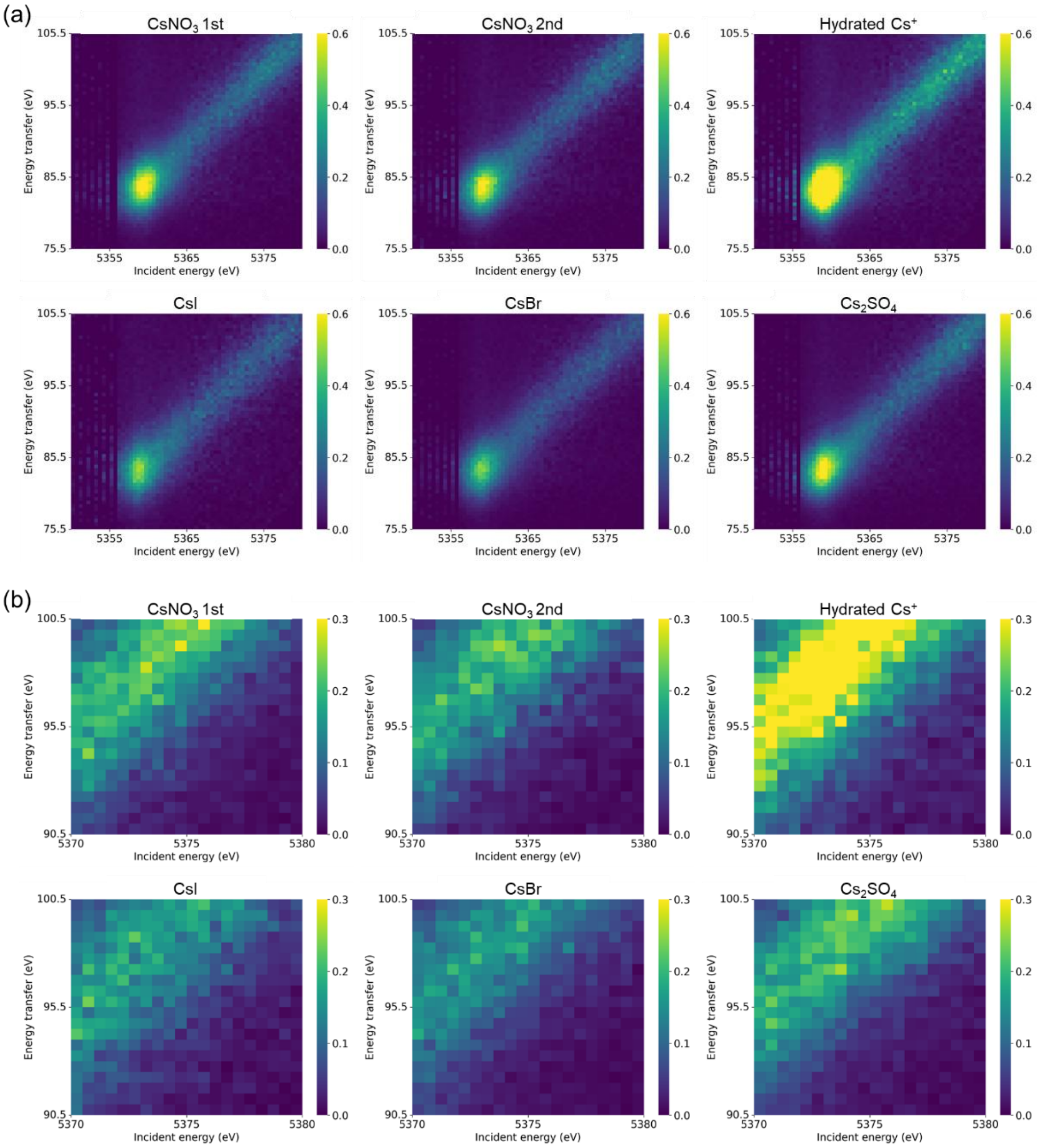

Figure S9 RIXS planes according to Lγ$_1$. The energy transfer was obtained by subtracting emission energy from incident energy. The color bar shows normalized intensity.
